# Disability pensions related to heavy physical workload: a cohort study of middle-aged and older workers in Sweden

**DOI:** 10.1007/s00420-021-01697-9

**Published:** 2021-04-20

**Authors:** Daniel Falkstedt, Tomas Hemmingsson, Maria Albin, Theo Bodin, Anders Ahlbom, Jenny Selander, Per Gustavsson, Tomas Andersson, Melody Almroth, Katarina Kjellberg

**Affiliations:** 1 Institute of Environmental Medicine, Karolinska Institutet, Stockholm, Sweden; 2 Centre for Occupational and Environmental Medicine, Region Stockholm, Stockholm, Sweden; 3 Department of Public Health Sciences, Stockholm University, Stockholm, Sweden

**Keywords:** Job-exposure matrix, Work ability, Retirement, Musculoskeletal, Cardiovascular, Respiratory

## Abstract

**Objectives:**

The aim of the study was to examine the associations between heavy physical workload among middle-aged and older workers and disability pension due to any diagnosis, as well as musculoskeletal, psychiatric, cardiovascular or respiratory diagnoses. The population-based design made it possible to examine dose–response and potential gender differences in the associations.

**Methods:**

About 1.8 million men and women aged 44–63 years and registered as living in Sweden in 2005 were followed regarding disability pension during 2006–2016, until ages 55–65 years. Mean values of physical workload and job control, estimated through gender-specific job-exposure matrices (JEMs), were assigned to individuals through their occupational titles in 2005. Exposure values were ranked separately for women and men and divided into quintiles. Associations were analyzed with Cox proportional-hazards regression.

**Results:**

The analyses showed robust, dose–response associations between physical workload and disability pension with a musculoskeletal diagnosis in both genders: the adjusted hazard ratio and 95% confidence interval for those with the heaviest exposure was 2.58 (2.37–2.81) in women and 3.34 (2.83–3.94) in men. Dose–response associations were also seen in relation to disability pension with a cardiovascular or a respiratory diagnosis, though the hazard ratios were smaller. Physical workload was not associated with disability pension with a psychiatric diagnosis after adjustment for job control.

**Conclusion:**

This study of the entire Swedish population of middle-aged and older workers suggests that higher degrees of physical workload may increase the risk of disability pension overall, and specifically with musculoskeletal, cardiovascular or respiratory diagnosis, in both women and men.

**Supplementary Information:**

The online version contains supplementary material available at 10.1007/s00420-021-01697-9.

## Background

Because of impaired health, many working-aged people are eventually excluded from the labor market and receive their income through the welfare system. With today’s aging populations and political goals to keep older people in the workforce, this is considered increasingly problematic (Eurofound 2015). Occupational groups show large differences in rates of sickness absence and disability pension (Piha et al. 2013; Ropponen et al. 2013; Samuelsson et al. 2013; Stattin and Jarvholm 2005), which indicate that certain aspects of work may be difficult to reconcile with illness and reduced capacity.

Heavy physical workload, including heavy manual handling, awkward body postures, repetitive and circulatory demanding work, seems to decrease the possibility of continuing to work up until an older age. Several studies have shown that disability pension is much more frequent among workers with heavy physical workload than in those without (Emberland et al. 2017; Ervasti et al. 2019; Friis et al. 2008; Halonen et al. 2019; Jarvholm et al. 2014; Karkkainen et al. 2013; Karpansalo et al. 2002; Kjellberg et al. 2016; Labriola et al. 2009; Lahelma et al. 2012; Prakash et al. 2017; Ropponen et al. 2014; Sommer et al. 2016). Other studies have shown that long-term sickness absence, which usually precedes disability pension, is also more common (Andersen et al. 2016; Sommer et al. 2016; Sundstrup et al. 2018). Furthermore, when older manual workers are asked, they specifically highlight physical workload as an occupational barrier and reason for early retirement (Andersen et al. 2019).

The associations between heavy physical workload and disability pension with a musculoskeletal or psychiatric diagnosis–the most common diagnoses–have been the subject of most previous studies and, in these, a clear increase in risk of disability pension with a musculoskeletal diagnosis has been shown (Ervasti et al. 2019; Karkkainen et al. 2013; Karpansalo et al. 2002; Kjellberg et al. 2016; Lahelma et al. 2012; Ropponen et al. 2014). Less frequent disability pensions, such as disability pensions with a cardiovascular (CVD) or a respiratory diagnosis, have not been possible to examine reliably in relation to physical workload (Karpansalo et al. 2002). However, these are diagnoses that likely impair performance in a job with heavy physical workload and, in this way, increase risk of early exit from the labor market through disability pension.

Previous studies have been limited by size and sometimes to certain occupations or labor-market sectors. Therefore, these studies have not been able to examine whether the rate of disability pension increases across the full range of physical workload. Few studies have been able to investigate this separately for female and male workers, although it is of interest as women and men are often relatively segregated in different sectors and occupational groups in the labor market. It is also of interest to investigate this association while simultaneously taking psychosocial working conditions into account. For example, heavy physical work is often co-occurring with small opportunities for workers to influence when and how tasks are to be performed (low job control), which has been linked to increased risk of labor-market exit through disability pension (Knardahl et al. 2017).

The purpose of this study is to contribute to an enhanced understanding of the association between heavy physical workload and early exit from the labor market through disability pension among aging workers. To achieve this aim, we studied the entire Swedish population of middle-aged and older working men and women. Hypothesized dose–response associations with all-cause and cause-specific disability pension were examined using a job-exposure matrix (JEM) for classification of physical workload, and also job control, across the full range of the workers’ occupations.

## Methods

### Study population, setting, and design

The study is based on the recently established Swedish Work, Illness, and labor-market Participation (SWIP) cohort, which includes all individuals 16–64 years of age and registered as living in Sweden in 2005, i.e. 5.4 million. We used a sub-population consisting of the 946,523 women and 888,032 men born during the years 1942–1961 and with occupational code available for the baseline year of the study, 2005. This corresponds to 76.9% of the almost 2.4 million individuals in total within the selected age range, which includes non-workers such as those already on early retirement at baseline. Follow-up regarding date and diagnosis of disability pension was made prospectively for the years 2006–2016. At baseline, the individuals in the study were aged 44–63 years; younger individuals were excluded, because, among them, disability pensions are less likely to be caused by work-environment factors.

### Data collection and variables

Data on occupations, classified on a four-digit level according to SSYK96 (Sweden’s classification of occupations based on ISCO-88), were obtained from Statistics Sweden’s ‘LISA’ register (‘Longitudinal integrated database for health insurance and labour market studies’) (Ludvigsson et al. 2019). The register starts in the year 1990 and contains information on every individual in Sweden aged 16–64, but occupational data are only available from 2005. The data on occupations were linked to the cohort and used for classification of work-environmental exposure using job-exposure matrices (see below for description of variables). Further data, used for multivariable analyses (age, gender, education, marital status, history of unemployment, and registered sickness absence), were obtained either from the LISA register, the Total Population Register at Statistics Sweden (Ludvigsson et al. 2016), or the Swedish Social Insurance Agency’s MIDAS register (‘Micro Data for Analysis of the Social Insurance System’, see forsakringskassan.se 2020a). An overview of the utilization of register data in the study is shown in Table S1.

#### Physical workload (exposure)

The study participants' degree of overall occupational physical workload was classified using a recently developed job-exposure matrix (JEM) for physical workload applicable to up to 355 occupations in Sweden. This JEM is based on data from the Swedish Work Environment Surveys 1997–2013, which in turn derives from eight questions regarding physical loads that involve heavy lifting, uncomfortable working postures, repetitive work and physically demanding work (Badarin et al. 2021). A gender-specific index mean value for the overall physical exposure, based on the eight questions, was obtained from the matrix and linked to individuals through yearly data on their occupations in 2005. Quintiles of physical workload were created based on the gender-specific mean values.

#### Disability pension (outcome)

In Sweden, disability pension (from the year 2003 termed ‘sjukersättning’, i.e. sickness compensation) is a compensation for those between ages 30 and 64 who will probably never be able to work full time due to illness, injury or disability (forsakringskassan.se 2020b). There must be a proven reduction in work ability by at least one fourth “in all jobs on the labour market”, including jobs that are arranged for persons with disabilities, such as employment with salary grants. Depending on how much the work ability is reduced, full, three-quarter, one-half or one-quarter disability pension may be paid. The Social Insurance Agency can initiate the process itself, but disability pension is otherwise applied for. Due to a policy change in 2006, the proportion of applications granted has been sharply reduced and is currently based on a thorough examination of the degree of work disability (Kadefors et al. 2019). If disability pension is granted, it can cover up to about 65% of lost income (in those with previous employment).

Data on disability pension (e.g. granting date, grade, and ICD diagnosis) were obtained from the above-mentioned database MiDAS. Virtually, all reimbursements related to disability pension are registered in this database. In the present study, disability pension diagnoses (according to ICD, 10th version) were as follows: musculoskeletal diagnoses, codes M00-M99; psychiatric diagnoses, codes F00-M99; circulatory system diagnoses, codes I00-I99; and respiratory diagnoses, codes J00-J99. All first-time disability pensions were included in the study, i.e., both full and partial.

#### Covariates

Level of education, marital status, and history of unemployment are considered as indicators of underlying employability problems and were obtained from the above-mentioned LISA register (Ludvigsson et al. 2019). *Level of education* was classified based on number of years of education: ≤ 9, 10–11, 12, and ≥ 13 years, which largely corresponds to compulsory school, vocational (high) school, upper secondary school, and university-level degree. *Marital status* was coded as married (or registered partner), unmarried/single, divorced, widow(er). *History of unemployment* was calculated from the total number of days with benefits during 5 years before the baseline, i.e. 2000–2004, and was divided into (1) no unemployment days, (2) 365 or fewer days of unemployment, and (3) more than 365 days of unemployment.

*Job control* was classified with a recently developed JEM on decision authority at work (using the same source of survey data as for the physical JEM). Four questions from the survey were used for the classification and measured respondents’ continuous/daily possibilities/opportunity to (1) decide when tasks should be done, (2) control the work pace, (3) take short breaks for talking, and (4) participate in and decide on the organization of own work (Almroth et al. 2021). Like the physical workload variable, a gender-specific index mean value for job control was obtained and linked to study participants through their occupational data. As for physical workload, quintiles of job control were created based on the gender-specific mean values.

### Data analysis

Among the 1.834,555 individuals, a minor proportion lacked information on level of education (816) and history of unemployment (221). Individuals with a disability pension before the start of the follow-up were excluded from the analyses.

To characterize the women and men with different degrees of physical workload, we calculated the distribution of covariates across the quintiles of physical workload. We also estimated the crude associations between covariates and all-cause disability pension.

To answer the question of whether and how heavy physical work may increase the rate of disability pension, we used Cox proportional-hazards regression, which estimates hazard ratios (HR). Person-time was calculated from January 1, 2006 up to the date of emigration, early (non-disability) pension, death, disability pension or the end of follow-up on December 31, 2016. Associations with disability pension due to any diagnosis or a musculoskeletal, psychiatric, cardiovascular or respiratory diagnosis were analyzed in crude and covariate-adjusted regression models (i, ii), and separately for women and men; the covariates were (i) level of education, marital status, and history of unemployment and (ii) plus job control. To investigate potential over-adjustment, we also carried out analyses stratified by education level and job control, respectively (both dichotomised).

Granting of disability pension is in most cases preceded by a longer period of sickness absence, and due to this structural relation, sickness absence before baseline was not included as a confounding variable. However, as a sensitivity analysis, we reran our analyses on a population excluding individuals with sickness absence registered at the Swedish Social Insurance Agency between 2001 and 2005.

We also checked whether the above-mentioned reduction in granted disability pensions, which was fully implemented by the year 2010, may have impacted on the associations. In this sensitivity analysis, physical workload was related to disability pensions (any) occurring in 2006–2009 and 2010–2016, respectively.

For calculation of HR attenuations (%) in multivariable-adjusted models, the following formula was used: (crudeHR-adj.HR)/(crudeHR-1)*100. In a previous study (Kjellberg et al. 2016), we found large changes in estimates after adjusting for confounding variables.

## Results

A characterization of the women and men with different degrees of occupational physical workload is seen in Table 1. In both genders, low levels of education and days of unemployment before the baseline year are clearly more common among those with higher degrees of physical workload. Marital status, however, differs most in men; those with more physical workload are more often unmarried or divorced at baseline. Lastly, among both women and men, having low job control is much likelier in jobs with higher degrees of physical workload than in jobs with low physical workload.

**Table 1:** Covariate distributions across degrees of physical workload

	Exposure to physical workload (quintiles)									
	Women					Men				
	Low	Low/mid	Middle	Mid/high	High	Low	Low/mid	Middle	Mid/high	High
*N*	184 192	197 541	178 327	197 040	189 423	176 150	177 823	176 113	181 068	176 878
	%	%	%	%	%	%	%	%	%	%
Education, years^a^										
≥ 13	69.9	38.6	62.9	10.3	7.4	68.4	46.4	30.2	7.8	5.7
12	9.1	15.2	7.0	9.8	10.9	14.3	20.3	17.0	11.6	10.9
10–11	16.0	34.1	23.0	54.1	55.0	11.8	22.5	32.3	42.0	44.9
≤ 9	5.1	12.1	7.1	25.9	26.7	5.6	10.8	20.5	38.6	38.6
History of unemployment (past years)										
No	89.9	84.8	87.5	77.9	81.1	91.0	88.5	83.5	79.5	74.9
< 365 days	7.4	11.1	9.6	16.5	14.5	5.7	7.7	11.3	14.2	16.0
> 365 days	2.1	3.7	2.5	5.1	3.8	2.2	3.0	4.7	6.0	8.6
Marital status										
Married or equivalent	60.7	62.8	60.6	59.8	57.5	70.0	66.9	59.3	54.2	53.5
Not married	16.8	15.7	17.5	16.4	18.1	15.7	17.3	22.3	27.3	28.9
Divorced	20.1	18.7	19.3	20.0	20.7	13.4	14.8	17.3	17.4	16.6
Widowed	2.5	2.9	2.6	3.8	3.7	1.0	1.1	1.1	1.1	1.1
Job control										
High	65.6	28.4	2.7	2.2	1.8	77.2	10.7	7.2	3.4	1.9
High/mid	17.2	42.4	16.3	2.7	19.6	13.4	51.4	10.7	14.7	13.3
Middle	12.7	10.8	47.2	13.5	12.5	4.4	15.5	24.8	13.7	38.4
Mid/low	0.4	0.2	18.7	58.0	45.5	4.9	5.2	31.1	47.2	16.8
Low	4.0	18.2	15.1	23.7	20.5	0.2	17.2	26.3	21.0	29.4

^a^ ≤ 9 years ~ Low secondary or shorter; 10–11 years ~ High secondary, vocational; 12 years ~ High secondary, academic, ≥ 13 years ~ university-level studies/exam

During the follow-up period, 71,057 (7.5%) women and 41,051 (4.6%) men were granted disability pension. In both genders, lower levels of education and more unemployment are associated with increased hazard of disability pension (Table 2). Furthermore, divorced and widowed women and men as well as unmarried men have an increased hazard of disability pension, compared to married individuals. Additionally, it is seen that low job control is associated with increased hazard of disability pension.

**Table 2:** Crude models of the associations between covariates and disability pension in women and men

Disability pension, any diagnosis	Women				Men			
	Cases		Crude model		Cases		Crude model	
	No	%	HR	95% CI	No	%	HR	95% CI
Education, years^a^								
≥ 13	21,831	6.21	1.00		8570	3.06	1.00	
12	7067	7.13	1.11	1.08–1.14	5474	4.17	1.46	1.41–1.51
10–11	29,032	8.33	1.42	1.39–1.44	14,580	5.34	1.78	1.73–1.82
≤ 9	13,127	8.91	1.71	1.68–1.75	12,427	6.11	2.19	2.13–2.25
History of unemployment (past 5 years)								
No	55,676	6.95	1.00		30,301	4.06	1.00	
< 365 days	11,700	10.4	1.41	1.39–1.44	7103	7.28	1.82	1.77–1.86
> 365 days	3681	11.22	1.63	1.57–1.68	3647	8.34	2.16	2.09–2.24
Marital status								
Married or equivalent	39,981	7.01	1.00		22,160	4.11	1.00	
Not married	12,020	7.53	0.95	0.93–0.97	10,229	5.16	1.20	1.17–1.22
Divorced	16,761	8.97	1.30	1.27–1.32	8130	5.76	1.41	1.37–1.45
Widowed	2295	7.75	1.41	1.35–1.47	532	5.66	1.67	1.53–1.82
Job control								
High	8865	4.68	1.00		4254	2.4	1.00	
High/mid	13,611	7.28	1.62	1.58–1.66	6949	3.78	1.59	1.53–1.65
Middle	14,518	8.09	1.76	1.71–1.80	9209	5.36	2.24	2.16–2.32
Mid/low	21,903	9.32	2.03	1.98–2.08	10,670	5.68	2.41	2.32–2.49
Low	12,160	7.81	1.72	1.68–1.77	9969	5.95	2.49	2.40–2.58

*HR* hazard ratio; *95% CI* 95% confidence interval

^a^ ≤ 9 years ~ Low secondary or shorter; 10–11 years ~ High secondary, vocational; 12 years ~ High secondary, academic, ≥ 13 years ~ university-level studies/exam

The crude regression models for the women (Table 3, upper part) show that higher physical workload is clearly associated with increased hazard of disability pension (any diagnosis). Furthermore, it is seen that disability pension with a musculoskeletal diagnosis, as expected, increases most in relation to degree of physical workload (HR = 4.38 for those in the category with highest exposure). Hazard of disability pension with a psychiatric diagnosis increases comparatively little while increases in hazard of disability pension with a cardiovascular, and especially a respiratory diagnosis, are clearly seen.

**Table 3:** Physical workload and disability pensions in the population of female middle-aged Swedish workers; crude and adjusted hazard ratios

Physical workload	Cases		Cox’ regression models					
			Crude		Adjusted^a^		Adjusted^b^	
	No	%	HR	95% CI	HR	95% CI	HR	95% CI
Any diagnosis								
Low	9258	5.03	1.00		1.00		1.00	
Low/mid	11,924	6.04	1.26	1.22–1.29	1.19	1.15–1.22	1.03	1.00–1.07
Middle	13,088	7.34	1.46	1.42–1.50	1.43	1.39–1.46	1.11	1.07–1.14
Mid/high	18,275	9.27	1.95	1.90–2.00	1.67	1.62–1.72	1.27	1.22–1.31
High	18,512	9.77	1.98	1.93–2.03	1.71	1.66–1.76	1.31	1.26–1.36
Musculoskeletal								
Low	1876	1.02	1.00		1.00		1.00	
Low/mid	3104	1.57	1.61	1.52–1.70	1.34	1.27–1.42	1.19	1.12–1.27
Middle	3830	2.15	2.09	1.98–2.21	1.97	1.86–2.08	1.62	1.52–1.73
Mid/high	7586	3.85	3.95	3.75–4.15	2.68	2.54–2.83	2.22	2.07–2.38
High	8382	4.42	4.38	4.17–4.61	2.97	2.81–3.14	2.45	2.29–2.62
Psychiatric								
Low	3912	2.12	1.00		1.00		1.00	
Low/mid	4488	2.27	1.12	1.07–1.17	1.15	1.10–1.20	0.97	0.93–1.02
Middle	4836	2.71	1.26	1.20–1.31	1.26	1.21–1.31	0.91	0.86–0.96
Mid/high	5004	2.54	1.23	1.18–1.28	1.27	1.22–1.33	0.86	0.81–0.91
High	4473	2.36	1.09	1.05–1.14	1.16	1.10–1.22	0.80	0.75–0.85
Cardiovascular								
Low	601	0.33	1.00		1.00		1.00	
Low/mid	753	0.38	1.19	1.07–1.33	1.09	0.98–1.22	1.00	0.88–1.12
Middle	747	0.42	1.28	1.15–1.42	1.23	1.11–1.37	1.06	0.93–1.21
Mid/high	1064	0.54	1.70	1.53–1.87	1.37	1.22–1.53	1.14	0.98–1.32
High	1123	0.59	1.82	1.65–2.01	1.47	1.31–1.64	1.23	1.07–1.42
Respiratory								
Low	115	0.06	1.00		1.00		1.00	
Low/mid	176	0.09	1.45	1.14–1.83	1.26	0.99–1.60	1.14	0.88–1.47
Middle	170	0.10	1.52	1.20–1.93	1.43	1.13–1.81	1.24	0.94–1.65
Mid/high	334	0.17	2.78	2.25–3.44	1.93	1.53–2.43	1.71	1.26–2.31
High	361	0.19	3.06	2.48–3.78	2.12	1.68–2.67	1.84	1.38–2.46

*HR* hazard ratio; *95% CI* 95% confidence interval

^a^Level of education, marital status, and history of unemployment

^b^Level of education, marital status, history of unemployment, and job control

In the models adjusting for differences in education level and unemployment history up to baseline (first adjusted model), it is seen that the hazard ratios for disability pension, all-cause and cause-specific, are attenuated, with the exception of disability pension with a psychiatric diagnosis. A calculation shows that approximately 50–60% of the associations between higher physical workload and disability pension with a musculoskeletal, cardiovascular or respiratory diagnosis remains, and approximately 70% of the association with all-cause disability pension. In the regression models additionally adjusting for differences in job control (second adjusted model), the hazard ratios are further attenuated: the increase in hazard of disability pension with a psychiatric diagnosis disappears; the increase in hazard of disability pension with a cardiovascular diagnosis is reduced by more than half; the increase in hazard of disability pension with a respiratory diagnosis almost by half–as is the increase in hazard of any disability pension. However, the increase in hazard of disability pension with a musculoskeletal diagnosis remains with 80% after this adjustment.

The regression models for the men (Table 4) show roughly the same pattern as for the women: after adjustment for education level and unemployment history up to baseline, the hazard ratios for disability pension with a musculoskeletal diagnosis are highest (crude HR = 8.17 and adjusted HR 5.02 for the category with heaviest workload); the hazard ratios for disability pension with a psychiatric diagnosis are lowest, and the hazard ratios for disability pension with a cardiovascular or a respiratory diagnosis or all-cause disability pension are in between. The attenuation of the hazard ratios was approximately 40–60%, with the exception of < 30% for psychiatric diagnosis. After further adjustment for differences in job control, this pattern remains, but with further attenuated hazard ratios (−50% for all-cause disability pension, −35% for disability pension with a musculoskeletal diagnosis, −100% for disability pension with a psychiatric diagnosis, and −40 to −50% for disability pension with a cardiovascular or respiratory diagnosis). The association with disability pension with a psychiatric diagnosis among men is eliminated after adjustment (−100%), in the same way that was seen among women.

**Table 4:** Physical workload and disability pensions in the population of male middle-aged Swedish workers; crude and adjusted hazard ratios

Physical workload	Cases		Cox’ regression models					
			Crude		Adjusted^a^		Adjusted^b^	
	No	%	HR	95% CI	HR	95% CI	HR	95% CI
Any diagnosis								
Low	4284	2.43	1.00		1.00		1.00	
Low/mid	5509	3.10	1.28	1.23–1.34	1.19	1.14–1.24	0.97	0.92–1.01
Middle	8580	4.87	2.05	1.97–2.12	1.73	1.66–1.80	1.29	1.22–1.35
Mid/high	10,701	5.91	2.49	2.40–2.58	1.90	1.82–1.97	1.40	1.33–1.47
High	11,977	6.77	2.86	2.76–2.96	2.11	2.03–2.19	1.56	1.48–1.64
Musculoskeletal								
Low	522	0.30	1.00		1.00		1.00	
Low/mid	973	0.55	1.86	1.67–2.06	1.59	1.43–1.77	1.33	1.17–1.51
Middle	1983	1.13	3.84	3.49–4.23	2.88	2.61–3.18	2.31	2.04–2.60
Mid/high	3358	1.85	6.35	5.79–6.97	4.03	3.65–4.45	3.23	2.86–3.65
High	4228	2.39	8.17	7.46–8.95	5.02	4.55–5.54	3.96	3.50–4.47
Psychiatric								
Low	1424	0.81	1.00		1.00		1.00	
Low/mid	1657	0.93	1.16	1.08–1.24	1.14	1.06–1.22	0.84	0.77–0.92
Middle	2448	1.39	1.71	1.60–1.83	1.60	1.49–1.71	1.04	0.95–1.14
Mid/high	2158	1.19	1.46	1.36–1.56	1.33	1.24–1.44	0.86	0.78–0.95
High	2386	1.35	1.64	1.54–1.75	1.45	1.34–1.56	0.94	0.85–1.03
Cardiovascular								
Low	693	0.39	1.00		1.00		1.00	
Low/mid	933	0.52	1.34	1.22–1.48	1.20	1.09–1.33	1.02	0.90–1.15
Middle	1334	0.76	1.96	1.79–2.15	1.56	1.41–1.71	1.26	1.11–1.42
Mid/high	1691	0.93	2.42	2.22–2.65	1.67	1.51–1.84	1.33	1.17–1.51
High	1621	0.92	2.37	2.17–2.59	1.59	1.43–1.75	1.28	1.12–1.45
Respiratory								
Low	70	0.04	1.00		1.00		1.00	
Low/mid	87	0.05	1.24	0.90–1.70	1.02	0.74–1.40	0.88	0.60–1.30
Middle	155	0.09	2.25	1.70–2.99	1.49	1.11–2.01	1.14	0.78–1.65
Mid/high	275	0.15	3.90	3.00–5.07	2.07	1.55–2.76	1.56	1.07–2.27
High	362	0.20	5.24	4.06–6.77	2.64	1.99–3.52	2.02	1.39–2.93

*HR* hazard ratio; *95% CI* 95% confidence interval

^a^Level of education, marital status, and history of unemployment

^b^Level of education, marital status, history of unemployment, and job control

Additional analyses showed that associations within strata of level of education and job control (low and high), respectively, did not differ from the non-stratified associations (results on all-cause disability pension in Tables S2, S3). Furthermore, excluding individuals with registered sickness-absence periods during a few years prior to baseline [160,653 (17.0%) women and 134,129 (15.1%) men] resulted in estimates similar to those seen in the main analysis (results on all-cause disability pension in Table S4). Also, associations with disability pensions occurring in 2006–2009 and 2010–2016, respectively, were similar (results on all-cause disability pension in Table S5).

## Discussion

Our analyses showed associations between occupational physical workload and disability pension with musculoskeletal, cardiovascular, and respiratory diagnoses. The association with disability pension with a musculoskeletal diagnosis followed a dose–response pattern and was the strongest of the associations. Associations remained in multivariable-adjusted models, including job control as a covariate. At the same time, we observed that adjusted estimates of the associations were often substantially attenuated as compared with unadjusted estimates. When differences in job control were adjusted for, no association between physical workload and disability pension with a psychiatric diagnosis remained.

### Comparison with previous studies

The association between heavy physical workload and disability pension with a musculoskeletal diagnosis has also been demonstrated in previous studies. A few of these studies had the opportunity to compare several different work-environmental factors and found that the risk estimates for physical workload appeared to be particularly high (Karkkainen et al. 2013; Lahelma et al. 2012). Most of the previous studies have been conducted in Finland and all have used a cohort design with several years or sometimes decades of follow-up data on disability pension. Study populations have varied: middle-aged men in Kuopio (Karpansalo et al. 2002), women and men in Helsinki (Ervasti et al. 2019; Lahelma et al. 2012), and Finish twins born in the first half of the twentieth century (Karkkainen et al. 2013). The sets of variables used for confounding control have also differed.

The largest gender-specific study on disability pension with musculoskeletal diagnoses before the present one is Swedish and was done on cohort data from just under 12,000 workers who were followed over a number of years in middle age with regard to disability pension and diagnosis (Kjellberg et al. 2016). For both women and men, the analyses showed a gradual increase in the risk of disability pension with a musculoskeletal diagnosis in relation to higher long-term physical workload. In multivariable models of the associations, childhood socio-economic position, late childhood cognitive ability, level of education, and degree of control at work were used for confounding control.

Associations between heavy physical workload and disability pension with diagnoses other than musculoskeletal have only been investigated in a small number of studies. One of these studies showed an association between heavy physical workload and increased risk of disability pension with a cardiovascular diagnosis (Karpansalo et al. 2002), although with a statistically uncertain risk estimate after multivariable adjustment. Thus, the present study goes further than previous studies by showing that disability pension with a cardiovascular or a respiratory diagnosis increases with higher degrees of physical workload in multivariable-adjusted models, among both women and men. We have not found any other studies that examined physical workload in relation to disability pension with a cardiovascular diagnosis. However, some studies have investigated disability pension with such a diagnosis in relation to psychosocial work-environmental factors, job strain (Mantyniemi et al. 2012) and effort–reward imbalance (Juvani et al. 2014) but did not show any robust associations.

In several of the studies referred to above, both crude and multivariable-adjusted estimates were reported, and we note that attenuation after adjustments was often large. In Karpansalo et al. (2002), the association between heavy physical workload and disability pension with a musculoskeletal diagnosis was greatly attenuated after adjustments for education level, BMI, alcohol consumption, smoking, maximum oxygen uptake and disease diagnoses at baseline and, in Lahelma et al. (2012), adjustment for occupational social class also had a considerable attenuating effect. In Kjellberg et al. (2016), the estimated hazard ratios were significantly smaller in models that adjusted for differences in late childhood cognitive ability, subsequent level of education, and job control, including disability pension with a musculoskeletal diagnosis. Similar effects were shown in the respective studies by Kärkkäinen et al. (2013) and Ropponen et al. (2014) of older twins, which could also adjust for differences in BMI, education, socio-economic position, marital status, and shift work. The present study adds to this by showing that the statistical association between higher physical workload and disability pension is further attenuated or even eliminated (for disability pension with a psychiatric diagnosis) when low job control is added to other covariates.

### Interpretation of the results

A decision at the Swedish Social Insurance Agency to grant disability pension is based on an assessment of full or partial long-term reduction of the individual's work ability (forsakringskassan.se 2020b). In turn, work ability is a function of both health and capacity of the individual and of the requirements and conditions in the individual's work, for example the degree of physical workload.

One likely explanation for the association between heavy physical workload and disability pension with a musculoskeletal diagnosis is that high biomechanical loading affects the musculoskeletal system negatively and decreases physical capacity. There is extensive evidence to support this explanation (da Costa and Vieira 2010; Punnett and Wegman 2004). Furthermore, the association with disability pension with a cardiovascular diagnosis may possibly be explained by overload and deleterious effects of prolonged and intense heavy physical work on the cardiovascular system, possibly involving exertion-related elevation of heart rate and blood pressure, and inflammatory processes manifested in arteriosclerosis (Petersen et al. 2012). However, there is a limited number of studies investigating the associations between heavy physical workload and cardiovascular diseases, and the results are inconsistent (Li et al. 2013; Petersen et al. 2012).

Another explanation may be that heavy manual occupations provide less opportunity for adaptations for older workers with physical illness and reduced physical capacity, which should have a bearing on disability pensions with both musculoskeletal, cardiovascular, and respiratory diagnoses. Furthermore, low decision latitude is a risk factor for disability pension even with musculoskeletal diagnoses (Lahelma et al. 2012). Conversely, occupations without physical strain are often non-manual occupations which allow more flexibility for the workers in terms of how and when tasks are to be performed (Johansson and Lundberg 2009). A hindering effect in physically strenuous jobs could give rise to the more general association with poor self-rated health (Burr et al. 2017), long-term sick leave (Andersen et al. 2016; Sommer et al. 2016; Sundstrup et al. 2018), all-cause disability pension (Emberland et al. 2017; Friis et al. 2008; Halonen et al. 2019; Jarvholm et al. 2014; Karpansalo et al. 2002; Labriola et al. 2009; Lahelma et al. 2012; Sommer et al. 2016) and, consequently, loss of work–life expectancy (Schram et al. 2020) demonstrated in previous studies. However, low education and a history of unemployment also seem to contribute to the explanation. We (Falkstedt et al. 2014; Kjellberg et al. 2016) and others (Schram et al. 2019) have shown in previous studies that the large amount of disability pension cases and loss of work–life expectancy among workers with physically heavy occupations may partly be explained by circumstances that relate to this group's weaker position in the labor market. It has also recently been shown on data from 26 European countries that the gap in work participation between workers with and without chronic diseases is substantially larger and increasing over time for workers with short education as compare to higher educational levels (Schram et al. 2019). To some extent, this could be related to limited opportunities for skills development and broadening of competence within manual occupations. Many years in a job that primarily involves routine task execution could lead to employment problems when health or physical capacity decline.

The lack of association with disability pension with a psychiatric diagnosis, after multivariable adjustment, indicates that physical workload does not create significant obstacles to work in the event of mental illness. The slightly higher prevalence of psychiatric disability pensions among women and men with physically heavy occupations seems instead to be explained by the co-occurrence of low job control in these occupations. There are some previous studies (see Knardahl et al. 2017) indicating that the probability of disability pension with a psychiatric diagnosis is increased at lower degrees of control at work.

### Strengths and limitations

One of the main strengths of the present study is the much larger size of the study population compared to all other previous studies focusing on a similar research question. This size allowed uniquely clear analyses of associations for disability pension with a cardiovascular or respiratory diagnosis, in women and men separately. The size and comprehensiveness also enabled analyses of the full range of exposure to physical workload in the labor market and, thus, hypothetical dose–response relations. Another strength is the use of JEMs for ranking of individuals within the study population in terms of physical workload and job control. The use of JEMs reduces differential misclassification and spurious risk estimates, which may arise from the use of self-reported data on physical workload and psychosocial factors at work (Fredlund et al. 2000). Initial differences in capacity and health do not affect the differences in physical and psychosocial workloads attributed by JEMs, as can be the case in self-reported data. An additional strength is the ability to account for the co-occurrence of physical workload and low job control.

Of course, using JEMs also has a downside. Through the construction of JEMs, differences in exposure between individuals within different occupations are eliminated, which can result in non-differential misclassification and downward bias of risk estimates. In addition, we know from previous research that questionnaire data on ergonomic factors, on which the JEMs are based, do not have the same precision as measurement and observation data (Teschke et al. 2009), but are the reasonable alternative for larger study populations. However, both recent (Madsen et al. 2018) and older studies (Bosma et al. 1997; Theorell et al. 1998) on physical workload or job-strain factors have found comparable results when using job-exposure matrices and self-reported exposures side by side. Furthermore, the JEMs in our study are indices that consist of mean value calculations based on multiple questionnaire items, which is a commonly used method in the field. An alternative is statistical modeling for the construction of JEMs, which Madsen et al. used recently and that possibly reduces misclassification to some extent. Finally, like these authors, we emphasize that analytical results based on JEM data refer to the occupational level and thus cannot be directly extrapolated to individuals.

We did not have access to data that could measure differences in lifestyle-related risk factors, such as tobacco smoking, physical inactivity, and obesity, and we cannot rule out the possibility of residual confounding. However, the studies that have had access to such information about the study participants have obtained approximately the same results as we did in this study, that is, remaining although attenuated associations. Furthermore, the adjustment for differences in age, education level, marital status, and history of unemployment before baseline should reduce potential confounding from lifestyle-related risk factors in the present study. As in other countries, low levels of education are known to be associated with several such factors (Falkstedt et al. 2016).

The multivariable models also did not include health status at baseline as a covariate. We chose not to adjust for registered sickness absence at baseline, as disability pension among workers is almost always preceded by long-term sickness absence, but the sensitivity analysis excluding individuals with sickness-absence periods in the years up to the baseline of the study gave results similar to those reported in the tables.

In conclusion, this study of the entire Swedish working population aged 44–63 years at baseline and followed for up to 11 years (censored at age 65) showed that, among both women and men, a higher degree of occupational physical workload is associated with a higher rate of disability pension with a musculoskeletal, cardiovascular or respiratory diagnosis. These associations, shown in multivariable-adjusted models, may reflect mechanisms reducing the chances of continued labor-force participation among older workers. An increased rate of disability pension with a psychiatric diagnosis was explained by confounding variables, including low job control.

## Supplementary Information

Below is the link to the electronic supplementary material.

Supplementary file1 (DOCX 24 KB)
