# Supplementary material for: Disability pensions related to heavy physical workload: a cohort study of middle-aged and older workers in Sweden
Source: Int Arch Occup Environ Health. 2021 Apr 20;94(8):1851–61. doi: 10.1007/s00420-021-01697-9 (PMC8490214; doi:10.1007/s00420-021-01697-9)
Supplement: Supplementary file 1 — Supplementary file1 (DOCX 24 KB) [file 420_2021_1697_MOESM1_ESM.docx]

| Table S1. Data and registers used in the study, calendar years concerned and the age of the participants during these years | | | |
| --- | --- | --- | --- |
| Data | Calendar years | Years of age | Register |
| Date of birth | 1942-1961 | 0 | RTB |
| Level of education | 2005 | 44-63 | LISA |
| Marital status | 2005 | 44-63 | RTB |
| History of unemployment | 2000-2004 | 44-63 | LISA |
| Occupational code | 2005 | 44-63 | LISA |
| Physical workload (JEM) | 2005 | 44-63 | JEM |
| Job control (JEM) | 2005 | 44-63 | JEM |
| Date of emigration | 2006-2016 | 45-65 | RTB |
| Date of death | 2006-2016 | 45-65 | RTB |
| Informational about early retirement | 2006-2016 | 63-64 | LISA |
| Disability pension with ICD10-diagnosis | 2006-2016 | 45-65 | MIDAS |
| JEM: Job-Exposure Matrix  LISA: Longitudinal integrated database for health insurance and labour market studies  MIDAS: Micro Data for Analysis of the Social Insurance System  RTB: The Total Population Register | | | |

| Table S2. Physical workload and disability pension (any diagnosis) in the population of middle-aged and older Swedish workers; hazard ratios estimated on women and men with low and high level of education | | | | | | | | |
| --- | --- | --- | --- | --- | --- | --- | --- | --- |
|  | Level of education, women | | | | Level of education, men | | | |
|  | Low | | High | | Low | | High | |
| Physical workload | HR^a^ | 95% CI | HR^a^ | 95% CI | HR^a^ | 95% CI | HR^a^ | 95% CI |
| Low | 1.00 |  | 1.00 |  | 1.00 |  | 1.00 |  |
| Low/mid | 1.11 | 1.05-1.16 | 1.23 | 1.19-1.27 | 1.02 | 0.95-1.09 | 1.26 | 1.20-1.32 |
| Middle | 1.37 | 1.31-1.45 | 1.44 | 1.40-1.49 | 1.52 | 1.42-1.62 | 1.78 | 1.70-1.87 |
| Mid/high | 1.67 | 1.60-1.75 | 1.55 | 1.49-1.62 | 1.62 | 1.52-1.73 | 2.29 | 2.16-2.42 |
| High | 1.70 | 1.63-1.78 | 1.64 | 1.58-1.72 | 1.79 | 1.68-1.91 | 2.62 | 2.48-2.78 |
| HR: hazard ratio; 95% CI: 95% confidence interval  ^a^ Adjusted for marital status and history of unemployment | | | | | | | | |

| Table S3. Physical workload and disability pension (any diagnosis) in the population of middle-aged and older Swedish workers; hazard ratios estimated on women and men with low and high job control | | | | | | | | |
| --- | --- | --- | --- | --- | --- | --- | --- | --- |
|  | Job control, women | | | | Job control, men | | | |
|  | Low | | High | | Low | | High | |
| Physical workload | HR^a^ | 95% CI | HR^a^ | 95% CI | HR^a^ | 95% CI | HR^a^ | 95% CI |
| Low | 1.00 |  | 1.00 |  | 1.00 |  | 1.00 |  |
| Low/mid | 1.14 | 1.10-1.17 | 1.14 | 1.11-1.18 | 1.02 | 0.98-1.07 | 1.04 | 1.00-1.09 |
| Middle | 1.35 | 1.30-1.40 | 1.44 | 1.40-1.49 | 1.46 | 1.37-1.55 | 1.45 | 1.38-1.53 |
| Mid/high | 1.42 | 1.34-1.51 | 1.37 | 1.31-1.44 | 1.83 | 1.73-1.95 | 1.72 | 1.63-1.81 |
| High | 1.85 | 1.78-1.92 | 1.88 | 1.82-1.95 | 1.55 | 1.47-1.64 | 1.98 | 1.89-2.08 |
| HR: hazard ratio; 95% CI: 95% confidence interval  ^a^ Adjusted for level of education, marital status, and history of unemployment | | | | | | | | |

| Table S4. Physical workload and disability pension (any diagnosis) in the population of middle-aged and older Swedish workers; adjusted hazard ratios estimated in Cox’ regression models *on individuals with no sickness absence in FK’s register 2001-2005* | | | | |
| --- | --- | --- | --- | --- |
|  | Women | | Men | |
| Physical workload | HR^a^ | 95% CI | HR^a^ | 95% CI |
| Low | 1.00 |  | 1.00 |  |
| Low/mid | 1.00 | 0.96-1.03 | 0.93 | 0.87-0.98 |
| Middle | 1.10 | 1.06-1.14 | 1.29 | 1.21-1.37 |
| Mid/high | 1.22 | 1.17-1.27 | 1.43 | 1.34-1.52 |
| High | 1.27 | 1.22-1.32 | 1.62 | 1.52-1.72 |
| HR: hazard ratio; 95% CI: 95% confidence interval  ^a^ Adjusted for level of education, marital status, history of unemployment, and job control | | | | |

| Table S5. Associations between physical workload and disability pension 2006-2009 vs. 2010-2016 (any diagnosis) in the population of middle-aged and older Swedish workers; hazard ratios estimated on women and men | | | | | | | | |
| --- | --- | --- | --- | --- | --- | --- | --- | --- |
|  | Women | | | | Men | | | |
|  | 2006 to 2009 | | 2010 to 2016 | | 2006 to 2009 | | 2010 to 2016 | |
| Physical workload | HR^a^ | 95% CI | HR^a^ | 95% CI | HR^a^ | 95% CI | HR^a^ | 95% CI |
| Low | 1.00 |  | 1.00 |  | 1.00 |  | 1.00 |  |
| Low/mid | 1.02 | 0.98-1.05 | 1.06 | 1.00-1.11 | 0.96 | 0.90-1.02 | 1.00 | 0.91-1.08 |
| Middle | 1.16 | 1.11-1.20 | 1.10 | 1.04-1.16 | 1.32 | 1.24-1.41 | 1.24 | 1.14-1.35 |
| Mid/high | 1.29 | 1.23-1.35 | 1.33 | 1.25-1.42 | 1.43 | 1.34-1.52 | 1.40 | 1.28-1.53 |
| High | 1.37 | 1.31-1.42 | 1.40 | 1.3-1.49 | 1.61 | 1.51-1.71 | 1.52 | 1.39-1.66 |
| HR: hazard ratio; 95% CI: 95% confidence interval  ^a^ Adjusted for level of education, marital status, history of unemployment, and job control | | | | | | | | |
